# Exploration of Psychological Resilience during a 25-Day Endurance Challenge in an Extreme Environment

**DOI:** 10.3390/ijerph182312707

**Published:** 2021-12-02

**Authors:** David Harrison, Mustafa Sarkar, Chris Saward, Caroline Sunderland

**Affiliations:** Sport Performance Research Group, The Sport, Health and Performance Enhancement (SHAPE) Research Centre, Department of Sport Science, Clifton Campus, Nottingham Trent University, Clifton Lane, Nottingham NG11 8NS, UK; mustafa.sarkar@ntu.ac.uk (M.S.); chris.saward@ntu.ac.uk (C.S.); caroline.sunderland@ntu.ac.uk (C.S.)

**Keywords:** endurance, extreme environments, mixed methods, resilience, stressors

## Abstract

Psychological resilience is the ability to use personal qualities to withstand pressure, consisting of the interaction between the individual and the environment over time. It is essential when operating in extreme environments which are typically characterised by a complex combination of stressors with increased elements of risk and adversity. Psychological resilience has never been investigated “live” (e.g., in the moment) throughout the duration of an extreme endurance challenge, despite anecdotal accounts of the need for resilience to successfully function in such environments. The aim of the study was to explore psychological resilience with challenge team members (*n* = 4, mean age = 46.0 years) involved in a 25-day extreme endurance challenge. The object of the challenge was to ‘TAB’ (Tactical Advance to Battle, fast marching with weighted packs) 100 peaks in the UK in 25 days and complete long-distance bike rides between base camps. A mixed-methods approach with a focus on qualitative methods was utilised. Specifically, individual reflective video diaries (*n* = 47) and focus groups (*n* = 4) were completed and analysed using interpretative phenomenological analysis (IPA). At the same time, the 10-item Connor Davidson Resilience Scale was employed to measure resilience, which highlighted the individualised and dynamic nature of resilience. Two superordinate themes were identified from the video diaries and focus groups, namely, the identification of the stressors within extreme environments and strategies to maintain functioning. Stressors were split into subordinate themes of significant and every day, and collectively, they created a cluster effect which contributed to pressure associated with operating in these environments. Challenge team members employed various strategies to maintain functioning, including using a challenge mindset to positively appraise pressure as a challenging learning experience. Further research should continue to develop an understanding of how participants completing challenges within extreme environments utilise and develop personal qualities to maintain functioning.

## 1. Introduction

The understanding of psychological resilience is inhibited due to theoretical and definitional difficulties [1] and remains an elusive concept in sport psychology [2]. Psychological resilience is complex [3], and conceptually, has been identified as a trait, process, or outcome to an adverse experience (for an in-depth review, see [4,5]). Historically, resilience has been defined as a trait which is a constellation of personal qualities that protect individuals from the potential negative effect of stressors [6]. These protective factors help individuals to withstand the pressure of the environments they are operating in [7]. However, this trait conceptualisation is outdated [5] because it does not take the environment, and how this interacts with the individual, into account [8], so scholars have suggested that resilience is a process [9] that is different within individuals and is more than just a fixed quality [10] and is, in fact, dynamic in nature [11]. Resilience has also been highlighted as context-specific [12], whereby individuals can show resilience in one environment but not another [10], and resilience changes over time [13] where individuals can show resilience at certain times but not others [13]. Resilience can cascade through time and is influenced by multiple processes across different systems [12]. Therefore, this process can be viewed as a “resilience bandwidth” [14], where individuals develop resilience over time based on their personality, environment, and the interaction between the two. This combined trait and process conceptualisation of resilience makes resilience an interactive concept between the individual and environment over time [10,13]. In line with this perspective, psychological resilience is defined here as “the role of mental processes and behaviour in promoting personal assets and protecting an individual from the potential negative effect of stressors” [15] (p. 675).

A grounded theory of psychological resilience in sport [15] was devised by interviewing twelve Olympic champions from different sports. This theory highlights resilience as a process and how the qualities of the individual interact and operate within the environment they are in. This grounded theory proposed psychological factors (e.g., positive personality, motivation, focus, confidence, and perceived social support) that influence how athletes appraise stressors in the environment as a challenge to influence their meta-cognitions. This process protects against possible negative effect of stressors experienced in elite competition. This protection is a result of individuals attempting to maintain homeostatic balance with internal and external stressors causing disruption at different bifurcation points [16] with the resources at their disposal. At these points, there is disruption where the individual attempts to reintegrate and re-establish balance within their homeostatic comfort zone. Recent work has highlighted this individual approach in terms of psychological flexibility in the COVID-19 global pandemic [12,17] with [18] suggesting that flexibility is the cornerstone of resilience. This flexibility increases psychological resource and is a combination of personal skills and qualities, which leads to increased resilience. Therefore, resilience is an individualised process with different people being affected by these stressors at different intensities and for different durations [13].

Consequently, the environment plays a significant role in resilience [14] pointing to the Ecology of Human Development model [19] being a suitable theoretical structure to explore the complex dynamic individualised nature of resilience. This model posits that there is interaction and influence from elements (e.g., experiences and people) within the immediate environment, but that there are also influences from other connected environments. For example, an expedition leader in an extreme environment has to contend with stressors in the environment (e.g., weather and terrain) but also stressors from other environments they are involved in (e.g., family and career). All these contribute to influence how the expedition leader will demonstrate resilience to function in the immediate environment. Additionally, The Everyday Stress Resilience Hypothesis [20] considers resilience through a systems perspective supporting the dynamic context of resilience [21]. This postulates that resilience increases from consistently experiencing stressors in different intensities and situations over time, very similar to the hay fever sufferer who takes a daily teaspoon of local honey to build inoculation to the pollen. Resilience is a result of an individual’s ability to manage stress and so their capacity to cope is increased. It has been suggested that resilience can initially protect individuals against adversity and then allow for positive adaptation to occur [4]. This indicates the interaction between the individual and the environment [8], whereby the individual has to appraise the adversity they face as a challenge [14]. This relates to the to the amount of challenge and support present in the environment, and for resilience to be demonstrated, there must be an element of adversity present which is typically present in an extreme environment [6]. This makes extreme environments the ideal setting to investigate psychological resilience.

Humans can operate in a wide range of environments and are capable of functioning within the extremes of these [3]. People will identify different environments as extreme due to their perceived individual capabilities to perform within them, so any environment that pushes an individual outside of their comfort zone can be considered extreme [22]. Examples of extreme environments are extensive with research being completed in space flight [23], expeditions in the polar regions [24,25], mountaineering expeditions [26], ocean crossings [27], and adventure/outdoor education programmes [28,29]. Therefore, extreme environments have been defined as settings that possess extraordinary physical, psychological, and interpersonal demands that require significant human adaptation for survival and performance [30].

Extreme environments are very complex and characterised by a range of intense stimuli [31], which people react differently to [23]. Examples of stressors within extreme environments are isolation, danger, risk, fatigue, sensory and social deprivation, and uncertainty [31], and therefore, they can be considered complex. Indeed, this complexity in the extreme environment of counter-terrorism operations has been explored [31]. This complexity can lead to a perceived lack of control among those immersed in them [30]. This may be in part due to a cluster effect, whereby the combination of these stressors can have a greater negative effect on performance as opposed to if individuals were exposed to individual stressors [31]. A review of the literature on working in Antarctica was completed [32] highlighting the stressors of operating in an isolated and confined environment (ICE), the consequences of living in such an environment (including anxiety and heightened friction, hostility, and conflict) and the coping strategies employed (e.g., preservation of group harmony, maintain schedules, and preparation pre-expedition). Therefore, the psychological response to these extreme environments is often the most important [3].

The contents of adventure education programmes (which are often conducted in extreme environments) typically possess similarities seen in individuals who demonstrate resilience [28]. These included perseverance, self-awareness, social support, confidence, responsibility to others, and achievement. Extreme environments encompass some form of risk and adversity that participants within them need to attend to so that they can positively adapt and show resilience [33]. The consequences of not being able to positively adapt can lead to serious injury or even death. This points to the importance of resilience in these environments [3], and to function in these environments, individuals have to demonstrate resilience [31], with numerous anecdotal accounts of the importance of resilience within extreme environments and a linguistic analysis of an individual within an Artic environment indicated that resilience might be demonstrated [34]. Despite this, research within extreme environments has mainly focused on the role of personality [26,27] and group functioning [25].

Over the past decade, psychological resilience has been investigated in sport (for reviews see [5,35]), with extensive research on resilience in athletes [36], teams [37], and coaches [38], with the majority of the studies investigating resilience using retrospective research methods [28] and single interviews at one time point [14]. However, these methods may not provide the depth of data required to adequately draw conclusions regarding the complexity of resilience due to recall bias and decay [35]. Thus, it has been suggested that resilience researchers should employ prospective and longitudinal qualitative methods to explore the process of resilience over time [5].

Surprisingly, psychological resilience has never been specifically investigated within an extreme environment, despite anecdotal accounts of the need for resilience to successfully function in such environments. Furthermore, no study has attempted to explore resilience “live” (e.g., in the moment) over time throughout an endurance challenge. Therefore, the aim of the study was to explore resilience “live” and longitudinally throughout the course of a 25-day challenge undertaken in an extreme environment.

## 2. Method

### 2.1. Research Design and Philosophical Underpinnings

A relativist ontological position was adopted for this study, as people construct reality and give meaning of specific phenomena (i.e., psychological resilience) in different ways, interpreting experiences based on their beliefs and past experiences to form their own notion of reality [39]. From an epistemological perspective, a subjective and transactional view of knowledge [40] was utilised due to the dynamic nature of the interactions over time between individuals and their environment, with the aim of the study to understand and interpret the world from the perspective of those involved (i.e., individuals completing a challenge in an extreme environment) [39], but also because of the complex [6] and individualised notion [10] of psychological resilience. This epistemological view makes the researcher an active member and inextricably linked with the challenge and the team members influencing the data collection and analysis [41]. This is known as the researcher as instrument, whereby the characteristics of the researcher may influence participants. The lead researcher of this paper visited the challenge four times, staying overnight in the base camp and completing tasks and interacting with both the challenge and support team to build a rapport [42]. However, by visiting the challenge, the researcher became connected to the challenge team members through their interactions, which may have influenced the research [41]. Hence, researchers must undertake self-reflexivity [41] and ensure methodological congruence [40], whereby all aspects of the methods are congruent with one’s philosophical standpoint.

In the present study, our interpretivist paradigm is congruent with the principles of Interpretative Phenomenological Analysis (IPA), chosen as the analysis of the qualitative data because it shows how individuals interpret their experiences based on their sense of reality over the course of the challenge [43,44]. The longitudinal nature of the study also aligns with the philosophical assumptions of IPA [45]. Longitudinal IPA has typically been used in nursing and health research [46,47], and IPA has previously been utilised in a sporting context investigating decreases in performance [36] and adventure psychology [48]. A key tenet of IPA is for the researcher to get as close to the data as possible to access the personal world of the participant taking an active role in what is a dynamic process of analysis [49], which was achieved by the lead researcher’s visits. This is because the interpretation process of IPA is double hermeneutic, whereby “participants are trying to make sense of their world; the researcher is trying to make sense of the participants trying to make sense of their world” [49] (p. 51). There also needs to be an understanding of the phenomenon in question (i.e., psychological resilience over time). Thus, for triangulation purposes, a quantitative resilience measure was utilised to track individual changes in resilience over time within the challenge team.

Researching in extreme environments presents unique practical challenges when completing research within them [3,50]. Specifically, there needs to be flexibility and simplicity in the research design to account for this complexity while maintaining quality data collection [50]. Therefore, creativity is needed when designing methods within extreme environments [3]. This creativity can be achieved by using mixed methods to provide better insights [51] and a holistic view of resilience [35] within extreme environments. Although research has typically tended to use a retrospective cross-sectional design, predominantly utilising quantitative methods, a mixed methods approach was employed to study resilience in an adventure education programme [28]. Incorporating qualitative methods into research completed in extreme environments can provide a significant contribution to the study of resilience by exploring the interaction of the individual within the complexity of an extreme environment [10] by producing a richer understanding of the subjective nature of psychological resilience [35,52]. This would elicit the depth needed to conceptualise and unpick the complexity and dynamic nature of psychological resilience live and longitudinally [5,52]. Indeed [34] also utilised a mixed method approach to provide insight into the psychological changes of participants before, during, and after an activity completed in an extreme environment, while [53] used mixed methods to explore stressors within a policing environment. Consequently, the current study employed a convergent mixed method research design [51,54] with an emphasis on qualitative methods. These qualitative methods explored the individual perceptions of the process of resilience within team members during the 25-day challenge. The quantitative methods allowed resilience to be tracked over time throughout the challenge to enhance the perceived individual changes identified in the qualitative methods.

### 2.2. Challenge Context

The challenge investigated in this study was the 100 Peaks Challenge [55] (Permission was granted by the organiser and 100 Peaks Challenge Team to name the challenge) completed for charity to create a legacy for the organiser’s younger brother, who was killed in action. The aim was to TAB (Tactical Advance to Battle), an acronym used by the British Military [56], up 100 of the highest peaks in the UK in 25 days. This is essentially fast marching with a weighted pack comprising essential kit (up to 30 lbs), which was deemed an extreme environment due to the significant stressors involved in completing the challenge. TAB marches are an essential component of most military training, where recruits are required to complete numerous weighted marches over various distances to simulate the pressures associated with battle scenarios [57]. Accordingly, there is a significant psychological aspect to this training [58] that has not yet been explored within the research. Alongside the TAB elements of the challenge, when the challenge team transitioned between locations, they cycled to the next base camp location which were situated in remote locations of the UK (for example, Snowdonia and Lake District National Parks), where the challenge team lived in tents, apart from the last location, which was a hotel a short distance from the last peak. At each location, a long-distance bike ride was also completed. Due to the extreme weather conditions during the challenge, some elements were either modified (e.g., TAB and bike routes adapted) or cancelled completely (e.g., Kayak across the Irish Sea to a peak on the Isle of Man) to ensure the safety of the team. During the challenge, a team of volunteers supported the challenge also living in each base camp. Furthermore, there were male and female ‘partials’ who completed routes with the challenge team, staying in base camp from one to several days.

### 2.3. Participants

In line with IPA guidelines, purposive sampling was utilised [49] to recruit a homogenous sample of 100% of the full challenge team (*n* = 4 total, *n* = 3 male, *n* = 1 female, mean age = 46.0 years, SD = 3.4 years). All members volunteered and had no obligation to take part signing an informed consent form before commencing the study. Due to the small challenge team, specific participant biographies have not been added to protect anonymity. The challenge team members had an average of 5.5 years (SD = 5.2) experience of completing TAB events. None of the team had completed a challenge of this magnitude before, but one was an army reservist and one was a former international cyclist. One was the organiser of the challenge. To maintain anonymity, gender neutral pseudonyms have been assigned to each participant.

### 2.4. Data Collection Methods

To explore psychological resilience “live” and longitudinally, multiple methods were employed in a mixed method approach with a focus on qualitative methods.

#### 2.4.1. Video Diaries

Video diary methods have been employed in outdoor adventure education settings [59] and extreme environments [34]. Due to the flexibility this method provides [60], video diaries allowed the complex individual narratives and experiences of psychological resilience during the challenge to be explored. Participants had autonomy with regards to content and duration of each video entry. Participants were given prompts attached to their video cameras to use as a guide if required (e.g., How has the day gone? What challenges have you dealt with today? How did you manage/deal with the challenges? What personal and/or collective qualities helped you to deal with the challenges?).

#### 2.4.2. Focus Groups

To stimulate collective team discussion around shared experiences during the challenge and explore the complexity of psychological resilience, four focus groups were employed [61]. The focus groups were completed on location in the base camps of the challenge (e.g., under a tarpaulin at the foot of Ben Nevis in Scotland). Focus groups have been employed as a method to investigate team resilience in a team setting to produce collective conversations and capture shared experiences [62]. Focus groups were chosen to explore the collective experiences of the challenge team.

#### 2.4.3. The 10-Item Connor-Davidson Resilience Scale (CD-RISC10)

The CD-RISC10 [63] was employed at four timepoints throughout the challenge. Psychometric evidence for the use of the CD-RISC10 to measure resilience has been provided in long-distance running [64] and cricket [65]. Psychometric evidence has also been offered in military populations [9]. Furthermore, elements of this measure have been utilised in adventure-based experiences [28]. Finally, the conceptual foundation for the measure originated in Shackleton’s experience of survival [66], making it a suitable measure to use in extreme environments. The CD-RISC10 is a 10-item measure with items such as “I am able to adapt when changes occur”, “Having to cope with stress can make me stronger”, and “I am not easily discouraged by failure”. Responses to each item are on a five-point Likert-type scale (0 = “not at all true” to 4 = “true nearly all the time”) [64]. The range of the total scale is 0 to 40, with higher totals indicating higher levels of resilience.

### 2.5. Procedure

Following institutional ethical approval, the lead researcher met the challenge team before the challenge started to complete the first baseline CD-RISC10. All subsequent data were collected on location while the participants were completing the challenge. Each participant was given an individual video camera and charger before the challenge started to record their video diaries. The cameras became their responsibility during the challenge. Participants were asked to find a quiet location to maximise confidentiality to complete their video diaries (e.g., participants tents, campsite bathrooms, and support vehicles used in the challenge). The completed recordings were collected by the researcher at the end of the challenge.

With regards to the lead researcher’s visits to the challenge, the exact days and times of these visits were organised during the challenge to minimise the impact of the research on the challenge [3]. During these visits, the focus groups were completed. The first three focus groups were recorded with members of the support team present as they doubled up as the challenge daily briefings. Additionally, the CD-RISC10 was completed by challenge team members at the same time as the focus groups and collected by the end of the visit. Finally, after each visit, reflexive notes were completed, and social media posts from the challenge and challenge team members were tracked.

### 2.6. Data Analysis

A total of 47 video diary entries were analysed, with each team member completing a varied number of video diary entries (Blair *n* = 10, Charlie *n* = 8, Jordan *n* = 12, Kendall *n* = 17). A total of 375 min of video footage (max length = 20:38 min and seconds, min length = 3:38 min and seconds, average length = 8:33 min and seconds, SD = 5:18 min and seconds) were collected from the challenge team video diaries. With regards to the focus groups (*n* = 4), a total of 97 min of data (max length = 29:50 min and seconds, min length = 17:40 min and seconds, average length = 24:40 min and seconds, SD = 6:09 min and seconds) were collected. The recording for each of the video diaries and focus groups was initially watched in its entirety to allow immersion in the data [67] and embodied transcription [68,69] was then utilised to transcribe the data. Embodied transcription is the process where the researcher is able to gain greater insights into the lived experiences of participants by speaking the contents of the video diary from the perspective of the participants into voice recognition software [69]. This made transcription an integral part of the data analysis as opposed to an initial act before interpretation [68] enhancing the ideographic aspect of the IPA process. The focus groups were transcribed verbatim.

IPA was used, as it offers a flexible analysis to identify central themes within the data [70] and differences within experiences across participants and over time to be shown [39]. After transcription, each transcript was read to get a ‘feel’ for the contents [49] and maintain an inductive approach [45,46]. Identification of initial themes were noted in pencil on the transcripts. Following this, each transcript was taken in turn and reread with further interpretation of the data and expansion of the initial notes into emergent themes in red pen. This was then transferred by handwriting extracts from the text and accompanying notes into a table of three columns (themes, original transcript, and exploratory comments). This process allowed further immersion into the data. The scoring of the CD-RISC10 was completed to give a resilience score for each participant at each time point. Descriptive statistics were completed at each time point to support the qualitative data to show individual changes in resilience over time. These were then presented diagrammatically to visually represent the change of resilience over time.

### 2.7. Methodological Rigour

Rather than universally applying rigour criteria, it has been advocated to select the most appropriate criteria [71] proposed by scholars [42,72] to ensure rigorous data that are fit for purpose for the specific research question. Specifically, to ensure rigour, the current study demonstrated prolonged engagement, persistent observation, and thick, rich description [42] of a worthy topic [72]. Rich rigour [72] was also ensured by considering the practical lessons learnt from [50] (i.e., completing a detailed planning process to ensure that the design had balance between simplicity while safeguarding the theoretical rigour of the process), and discussing the process with critical friends [71]. To ensure that the IPA analysis was of the highest possible quality, IPA research markers highlighted by [73,74] were utilised. Namely, a compelling narrative of resilience over time within the 100 Peaks was provided to emphasise experiences and factors important to the participants pertinent to understanding resilience in an extreme environment. This was done by spending time to consider and interpret the choice of words used by participants, in line with the double hermeneutic interpretation of IPA. In line with recommendations by [71], member reflections were completed with two of the challenge team to ensure philosophical coherence and rigour of the research. There are often misunderstandings around the generalisability in qualitative research because the statistical processes to achieve generalisability in quantitative research are not applicable, so different criteria need to be used for qualitative research [75]. The current study utilises naturalistic generalisability whereby the research resonates with the reader’s perceptions of expe-riences they have had, allowing them to reflect on the experiences of the challenge team and make connections with their own life experiences [75]. The epistemological assumption of the research also allows transferability, which is another criterion of generalisability [75]. As knowledge is constructed by the perceptions of the individual and is so subjective in nature, the reader can identify what is similar to their own experiences that can be generalised to other contexts. To achieve this, the research has utilised rich and detailed extracts from the challenge team and provided interpretative richness to assist the reader to think about the results and how they connect with their own experiences and transferred to other contexts. Reflexivity is an important feature of qualitative research [76] and IPA [77], since the researcher needs to understand their role in relation to others and to ensure rigour in the data collection process [78]. Therefore, the lead researcher went through a process of reflexivity to become aware of how they might have influenced the experiences of the challenge team during their visits. This was to increase awareness of potential subjective preconceptions derived from their identity as a neophyte PhD researcher invited to complete the research by the lead organiser of the challenge and background in sport psychology. This process also provided a valuable perspective of the individual and longitudinal nature of psychological resilience within an extreme environment.

## 3. Results and Discussion

**Figure 1:**
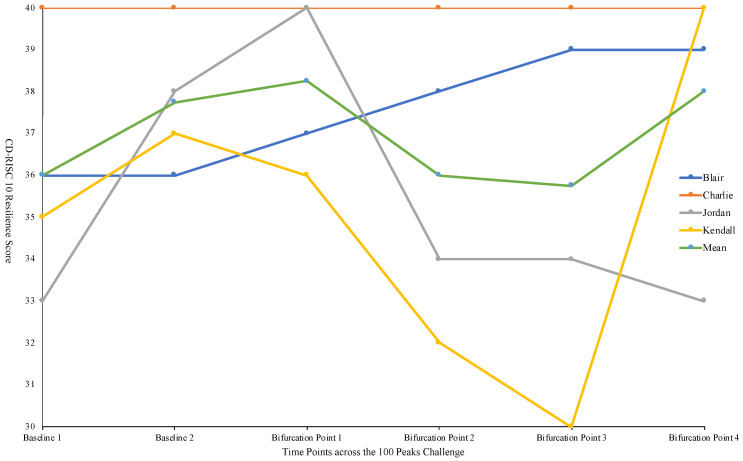
The individual CD-RISC10 scores of challenge team members across the time points of the 100 Peak Challenge. The time points of the challenge where the data were collected was determined by the Challenge Team, the Support Team, and the conditions at the base camps. Hence, they were not standardised. Below is some additional information to add context to these time points in the graph: Baseline 1—University in London, England. Completed 3 weeks before the challenge started. Baseline 2—Fort William, Scotland. Completed 24 h before the challenge started. Bifurcation Point 1—Fort William, Scotland. Completed on day 1 of the challenge. Bifurcation Point 2—Newton Stewart, Scotland. Completed on day 7 of the challenge. Bifurcation Point 3—Keswick, England. Completed on day 14 of the challenge. Bifurcation Point 4—Merthyr Tydfil, Wales. Completed on day 24 of the challenge.

The results of the current study highlight the individualised, complex, and dynamic nature of psychological resilience within extreme environments. Specifically, two superordinate themes were identified, the identification of the stressors within extreme environments by those operating within them and how these are perceived and influence an individual’s ability to maintain functioning over the duration of an ultra-endurance challenge. Furthermore, challenge team members employed various strategies to maintain functioning within the extreme environment. Taking both of these superordinate themes into account, this section will begin with an overview of the stressors in the 100 Peaks Challenge because the relationship between the individual, their interaction, and the environment cannot be researched independently [14], as there is a need to identify and understand the unique stressors within the environment (e.g., when they appear, their duration, and their frequency) when resilience is being investigated. The context in which resilience is demonstrated is important [13] because the process has context sensitivity with strategies being employed varying across individuals and environments [79]. The results from the video diaries and focus groups highlighted that the 100 Peaks environment was complex and extreme with an array of stressors with six subordinates identified (see Table 1). These stressors were split into significant and every day, which created a cluster effect [31] that influenced an individual’s ability to maintain functioning in these environments. Individual changes in resilience over time were identified by the results from the CD-RISC10 (see Figure 1), suggesting that the cluster effect influenced each individual differently at different times throughout the challenge, because there is individual variability in the appraisal of the stressors in the environment [13]. The reflective accounts of the challenge team highlighted this variability and also identified the significance of when the cluster effect started and how this subsequently cascaded through the reminder of the challenge. Following this identification of stressors, an exploration of how these stressors were perceived and influenced individuals’ resilience throughout the course of the 100 Peaks Challenge will be presented focusing on how team members maintained their functioning (see Table 2); three subordinate themes were identified, namely using a challenge mindset, which included accepting the environment, putting one foot in front of the other, and the use of humour. The complexity of social support and how it was used as a strategy will also be discussed alongside the influence of interpersonal differences.

### 3.1. Identification of Stressors during the Challenge

The 100 Peaks Challenge had numerous stressors and challenges that needed to be overcome. These changed depending on the location and time point within the challenge. For example, base camp 1 had high temperatures and high numbers of midges, where base camp 3 had high levels of wind and rain. Each stressor had to be perceived by the challenge team members individually [23] so that they could adapt to the stressor and maintain functioning. Therefore, the amount of time this took was different in all team members. This could account for the variation in the CD-RISC10 scores recorded during the challenge.

The stressors identified during the challenge were split into significant stressors and everyday stressors. Significant stressors were further split into personal administration errors (e.g., getting lost while on the mountain, wrong kit/nutrition strategies) and unpredictable disruptive incidents [15] (e.g., becoming ill, bike crashes, transition between base camps). Personal administration errors typically affected the participants more than unpredictable disruptive incidents, which were accepted as part of completing the challenge in an extreme environment. These stressors had serious consequences for the challenge team, as each member needed time to perceive and then comprehend each of these stressors to initiate appropriate strategies to maintain functioning. Unpredictable disruptive incidents were events that were out of the control of the challenge team. Furthermore, stressors either had a direct or indirect effect on challenge team members [80], as it became apparent that the interpretation of stressors by others and their subsequent response/actions to them had complex and varied consequences impacting the rest of the challenge team. During the challenge, this actually became another stressor affecting the day-to-day ability to complete daily tasks. Although part of the challenge and predicted, transitions between base camps could be considered a significant stressor because these transitions had a disruptive effect and contributed to the cluster effect (see below).

Everyday stressors were more challenge- and environment-specific. These were generally more accepted as part of being in an extreme environment and completing such a challenge. These included weather, terrain, and other aspects within the environment. Being exposed to these stressors contributed to the challenge team members’ overall comfort and strategies had to be employed to maintain functioning. For example, Blair and Kendall highlighted the extremely hot conditions early in the challenge by saying:

“Some serious terrain challenges, makes you question the choices, choices you are making. Obviously, the heat today, apparently it was the hottest place in the UK today and we certainly felt it. So, obviously, that adds some real complexity to being out on the hills.”

“It was a very hot day and a very tough day…the first few hills going, I could feel my quads and my calves but I expected that so I went through and we just managed through the day.”

#### 3.1.1. Cluster Effect

The results of the current study suggest the presence of a cluster effect of stressors within the extreme environment. Challenge team members had to withstand the effect of a range of stressors to ensure the challenge objective was achieved [81] and it is the clustering effect of these stressors that may have caused reduced functioning [30]. Each individual stressor chipped away at the challenge team’s capacity to function where their effect compounded as they clustered together. Blair summarised this by stating:

“Today, it’s just knowing what’s required in these sorts of conditions to ensure you are safe, the group, the people you are with, the team are safe, are they doing the right things…with the cumulative effect of what we’re doing.”

This cluster effect has been evidenced in other research completed in extreme environments. Individual stressors can be tolerated, but when they cluster, the potential negative impact on performance can be increased [31]. This cluster effect can be thought of by considering the metaphor used by Jordan, who suggested the extreme environment was “a bit of a pressure cooker environment; you’re spending a lot of time with people that you don’t know particularly well, some you know better than others” when thinking about the effect different stressors have and how they interact as well as how people perceive and deal with these. The cluster effect works by inhibiting the individual’s ability to recover from stressors, which subsequently could cause acute stressors to develop into chronic stressors. For example, Blair provided evidence of the complexity and the individual nature of perceiving the cluster effect in relation to personal administration errors and unpredictable disruptive incidents focusing on the social dynamic (see below) within the challenge during the third focus group.

“But I think obviously we’ve had it difficult, the support team have had it difficult because it’s been there are challenges within the challenge no one anticipated that we would have to deal with the midge infestation that we had or well just the biblical efforts of the weather. And of course that puts a strain on things. It’s natural you’re isolated because you can’t actually sit and be a complete community cause the only shelter is a piece of polythene. We’re in each other’s pockets 24 h a day, 7 days a week with people that you’ve not lived with spent time with and we’ve all got our own idiosyncrasies. We’ve all got our own ways of doing things and at time yeah you do lose it. You don’t lose your temper or you get moody or whatever and take yourself away from it but then in the end you’ve just gotta come back and carry on.”

#### 3.1.2. Different Stages and Bifurcation Points

Each extreme environment has a unique variety and combination of stages [80] that individuals must work through. Within the resilience literature, [15] coined the notion of bifurcation points to discuss resilience within a life span perspective. Bifurcation points can be applied to an extreme environment as the ability to survive, function, and perform within these requires adaptation over time to deal with the array of stressors present in the environment before moving onto the next bifurcation point. However, this is not always possible, causing a cascade effect during the challenge. This indicates that if the stressors cascade, so does resilience, as this process buffers against the impact of these stressors [12]. With regards to the 100 Peaks Challenge, the four time points or bifurcation points in this research represent four different base camps and the transition between them. Consequently, each contained different combinations of stressors in different sequences than other timepoints for each challenge team member to deal with and process.

Stressors ranged in number and severity depending on the bifurcation point that the individual was in, and how they perceived these stressors. These stressors can build up creating a cluster effect if a longer time to adapt is needed to overcome them. Hence, stressors can build up within and between bifurcation points, producing a more intense cluster effect, as individuals have to attend to existing stressors (from previous bifurcation points) as well as adapting to a new combination of stressors in the subsequent bifurcation point. This is emphasised by Kendall during the last focus group the night before the final day:

“It is an unusual environment to be in and you all get tired you all have good days and bad days and you get through that. There’s not a lot of choice you just focus on the next day but there’s always something coming next or you have to get ready or there’s like people asking questions or prepare for the next day it’s all go, go, go. And I think it’s for everyone it’s for the support team it’s also for us the same…Yeah so we didn’t really have a lot of time to actually chill and relax and let things sink in because we had long days and we had to get ready for the next one and think about the next day so we didn’t have a lot of like downtime just to chill and just sit as a team. And also because of the midges and the weather we kinda stuck in our tents sometimes so yeah.”

#### 3.1.3. Start of the Cluster Effect

The transitions were planned and an integral part of the challenge but became unpredictable disruptive incidents, as they were not expected to have the effects they had on the challenge team. This first transition from base camp 1 to base camp 2, where challenge team members moved from bifurcation point one to bifurcation point two (see Figure 1 for challenge team members’ individual CD-RISC10 scores) within the challenge, was a highly significant time point, as it created a unique combination of stressors that allowed the cluster effect to be observed for the first time. This is because the full impact of moving the kit, the logistics of changing location, and the subsequent knock-on effects of this process to challenge team members were not foreseen. Additionally, transitions presented an increased risk due to the challenge team cycling between base camps often in extreme weather conditions as well as the support team moving all the kits.

The context of this transition is important as it became a critical point of the challenge for all participants. The team experienced some significant stressors (both personal administration errors and unpredictable disruptive incidents, see Table 1 and identification of stressors section) at base camp 1 and there was an expectation that things would improve with regards to these. The lead researcher witnessed the team arrive from a long cycle 107 miles in high spirits, but this quickly dissipated with the realisation that the transition from a logistics perspective had been delayed and the camp was still being constructed. The lead researcher arrived at the base camp location before the support team, as they were delayed in traffic. When the support team arrived, the lead researcher assisted in constructing the base camp and completed the second focus group a couple of hours after the challenge team’s return. Additionally, there were a large number of midges present at base camp 1 and there was hope that moving south would see the number of these decrease. However, the reverse was true, and the number of midges actually increased. This increase in midges, base camp 2 not being ready clustered with other stressors such as fatigue, and differing levels of fitness at this bifurcation point made the transition to base camp 2 very difficult for the challenge team. Kendall attempted to articulate this difficulty in relation to the cluster effect:

“Sure like it’s if you go to find the cycling and the climbing the peaks like Jordan has said as well and the weather and the midges and just everything else even like transition day packing up and things like that those are challenges on their own apart from the cycling and you’ve got challenges also getting along with the team and making sure everything work well the support team those are separate challenges on top of what we do already so and yeah.”

And Charlie articulated the experience:

“The day went pretty well. Coming back to base camp, talking to the [support team] they have had a nightmare setting up the base camp. The midges here at the moment are absolute hell. That is, one of the hardest challenges is dealing with the midges.”

Additionally, stressors from pre-challenge bifurcation points may not have been dealt with by challenge team members and came to the fore as stressors clustered together. This was particularly evident for Blair, who struggled with the reasons some of the challenge team members were completing the challenge:

“I’ve found some situations difficult to deal with purely because I don’t understand the mentality of certain individuals that they make, and, because of the nature of this challenge. Because of the nature of the physicality of it and everyone is getting tired the demands are great and conditions aren’t ideal. Small things become big things.”

The appearance of the cluster effect at this point was significant as the challenge team members never really seemed to recover from the stressors from this transition, which compounded further stressors, as they were dealing with stressors from this transition as well as those present in subsequent bifurcation points. This could be due to physiological stressors (e.g., fatigue and muscle soreness) having psychological manifestations that add to the burden of individuals [82]. Jordan talked about the particular transition and the impact it had on time, causing a video diary to be missed, which was caught up with the following day:

“And because we had the transition before. It was a late start which wasn’t ideal but it is what it is so we cracked on with the TAB and immediately found that the ground was quite tough.”

This was also the case with Blair, “It’s just been a little mad with the transition and the last couple of days”. It could be described as the tipping/breaking point within the challenge with regard to interpersonal differences (see subsection below).

### 3.2. Exploration of Resilience

To make sense of the ‘lived experience’ of the challenge team [44] and to understand the meaning they prescribed to these experiences [49] from a resilience perspective, key themes from the participant’s narrative were identified with similarities and differences between them highlighted in each theme [39]. Each individual perceived their experiences in a different way, evidenced in the variability in the CD-RISC10 scores between participants at each bifurcation points, and the nuances of these differences were exhibited in their respective narratives in the video diaries. Within the focus groups, these differences were less pronounced. This suggested the individualised and dynamic nature of psychological resilience throughout the challenge, supporting the notion that resilience is a process [9] with interaction between the individual and their environment [13]. The individual CD–RISC10 scores depicted each member of the challenge team demonstrated a different pattern of their resilience scores across the bifurcation points, indicating that an individual’s resilience is context-specific within the different stages of a challenge [12]. The CD-RISC10 scores also indicate that challenge team members had different levels of resilience entering the challenge, and the trajectory of each team member was different during the challenge, indicating that resilience is an individualised process that changes over time. Charlie scored full points across every time point demonstrating they had high levels of resilience. This was reinforced during their video diaries and their comments in the focus groups, suggesting they had the necessary resources to deal with the environment. This also highlights the importance of using mixed methods to explore complex phenomena such as resilience, as this may have been missed using only one method. Jordan had their highest score at the first bifurcation point, then showed a sharp decrease near the end of the challenge, indicating that they were still contending with the cascade of the cluster effect through the challenge. Kendall showed decreases in psychological resilience throughout the first three bifurcation points before experiencing a large increase at the final time point. This could demonstrate that the challenge had a positive effect on their resilience, and they had worked through the cluster effect and developed the necessary resources to cope. It could also indicate that the end of the challenge was a key focus. Finally, Blair showed a steady increase in their resilience during the challenge, showing that their trajectory throughout the challenge was positive and that they were able to deal with the cluster effect and developed the strategies to effectively function. These individual changes in psychological resilience will now be explored through the strategies used by the challenge participants to highlight the complexity and dynamic nature of resilience over time.

#### Challenge Mindset

Appraising experiences and stressors as a challenge rather than a threat is known as a challenge mindset [14] and was used throughout the challenge by all the participants. Individuals evaluate and appraise the stressors in the environment against the resources they have at their disposal seeing difficult experiences as an opportunity for growth [15]. This challenge mindset can be split into three further subordinate themes of acceptance, putting one foot in front of the other, and humour. Each is used to reappraise stressors into a challenge; each sub-theme will now be discussed.

*Acceptance.* Acceptance of the conditions within the extreme environment was used consistently by all participants throughout the challenge and could be seen as challenge appraisal [14]. This was seen in the interpretation of some of the language used within the video diaries, a way to achieve excellence in IPA [73]. Words such as “Brutal”, “Tough”, and “Ridiculous” were often employed to describe the weather conditions and terrain. For example, Charlie stated, “…I mean every single mountain we’re going up it’s ridiculous, the incline of it and everything, it’s just”. These words were often used while highlighting that the experience was enjoyable and fun suggesting this form of acceptance had a masochistic element. They all seemed to enjoy this aspect of the challenge and had accepted this as part and parcel of completing the challenge.

This was evident for Charlie, who enjoyed the challenge of being in the mountains. They were less favourable about the cycling elements of the challenge but accepted them as part of the challenge and as something to get through so they could get back in the mountains.

“It was a big challenge for me yesterday. I haven’t covered anywhere near that distance…another challenging day, but the hills are why I am here. The cycling isn’t my thing. It’s something I’m just doing [to get back in the hills].”

This acceptance was also shown in how team members sometimes showed an inability to articulate verbally their perceptions of the cluster effect and its impact. This difficulty to articulate may be a demonstration of acceptance of the situation, simplifying the experience to just putting one foot in front of the other (see below) to get the job done while appraising the situation as a challenge. Jordan highlighted this difficulty in one of their video diaries:

“The main challenge was the terrain for us from that point of view, absolutely horrendous again. It is so hard to explain what you have to go through to get to the top of the mountains.”

Achieving a complete reflection of the cluster effect may take time, as individuals are first concerned with getting the job done, and then must reflect and appraise later as they digest what has happened. For example, Blair found it difficult to articulate the stressors within the environment. They also found it difficult to do this with the qualities and strategies they exhibited during the challenge to demonstrate resilience. Additionally, as Blair progressed through the challenge, their appraisal of their experiences showed they began to accept the cluster effect, which increased their resilience over the duration of the challenge, as evidenced by their CD-RISC10 scores. This could show that challenge appraisal is an ongoing process and the experiences within the challenge allowed them to find meaning from having to deal with the cluster effect as well as with the personal circumstances that brought them to the challenge. The reasons why they were completing the challenge carried more significance than short-term discomfort of the stressors from the extreme environment. As Nietzsche highlighted “He who has a why to live for can bear with almost any how”, and this was seen with Blair as they accepted their circumstances to find meaning:

“In comparison to what this time of year means to me, this challenge isn’t anywhere near as tough as what this period of time means. So again, being away from home, maybe that’s more of a challenge than this is.”

This ability to find meaning through the acceptance of their circumstance allowed individuals to transcend beyond the stressors they have deal with [83] to complete the daily challenges they were faced with and get something out of the experience.

*Putting one foot in front of the other.* To combat the cluster effect all participants spoke about “putting one foot in front of the other” to reduce the impact of the stressors in the cluster effect. This allowed participants to break down and concentrate on relevant stressors to maintain functioning to achieve daily objectives. Perseverance was identified as a key element [28], which could be considered similar to putting one foot in front of the other. By focusing on the next step, team members maintained the challenge mindset to combat stress while persevering with the objective of completing the challenge. This is highlighted by Blair in one of their video diaries:

“The terrain, its actually very very difficult to make people understand unless you’re doing these routes how demanding actually those trails are. So, that’s a challenge and the only way you can deal with that challenge is putting one foot in front of the other…I don’t stop. I keep going. I keep focused on what we are trying to achieved.”

And Jordan also highlighted this notion of focusing on the next step, “you keep going keep focused you don’t really think ahead a lot other than the next step you have to make or the next…”.

All participants highlighted how tough the conditions were but there was a need to get the job done and to keep moving by putting one foot in front of the other. This could be considered a challenge mindset, which [14] have highlighted as a major feature of resilience, whereby individuals positively perceive the stressors they face and the resources they have at their disposal as positive. It allowed challenge team members to develop acceptance, allowing them to accept the tough conditions (e.g., everyday stressors such as the weather and terrain) as part of completing a challenge in an extreme environment. However, personal administration errors of challenge team members were harder to accept. This could be due to the potential consequences of these for individuals and the wider challenge. During the challenge, these administration errors could not be dealt with, and they contributed to heightened levels of stress in base camp. It has been highlighted that distancing and removing yourself from a situation is a useful resilience strategy [16], but this could not be done during the challenge due to the base camp environment being an isolated and confined environment [30].

This challenge appraisal allowed challenge team members to employ specific short-term targets to focus on (e.g., putting one foot in front of the other to get to the top of the mountain etc). This kept them in the present and allowed them to keep moving without becoming overawed by the magnitude of the challenge and the cluster effect of stressors within it. Charlie encapsulated this by saying:

“Mentally its draining purely because every single step we’re taking, especially on the ridges, every single step you’re taking you’re having to constantly watch your footing and that is taxing.”

By completing these short-term targets, it brought the long-term objective closer to being achieved and gave them some semblance of order and control over a complex and uncontrollable environment, allowing the extent of the challenge to be cognitively reappraised. The ability to reappraise and show psychological flexibility adjusted the behaviour of individuals so that the long-term goal could be achieved [18].

Despite this short-term focus, challenge team members were also acutely aware of the long-term objective of completing the challenge and getting the job done. This was very business-like, with an external focus strategy utilised especially in the early stages of the challenge. Their focus was on the support team as opposed to using an individually and internally focused strategy. Charlie and Jordan bestowed praise on the work the support team were undertaking. This stopped as the cluster effect cascaded through the bifurcation points of the challenge and participants reverted to “putting one foot in front of the other”.

This challenge appraisal also allowed them to maximise safety and appraise risk correctly on the mountain while still balancing the requirements of achieving the objectives of the challenge ensuring the safety of everyone involved. This was because they were not taking up resources trying to deal with stressors presented in the environment. This risk appraisal was important so that undue risk was not taken at the expense of increasing the probability of an injury just to complete the challenge, while in the mountains, the challenge team had a responsibility to each other to ensure safety and this kept them going, supporting the work of [28]. This was a fine balancing act that required the situation to be constantly appraised while objectively taking into account the resources available to team members. This was done with honest objective communication in the form a ‘Chinese Parliament’ (a term used by the challenge team to describe a completely open and honest forum which is used in the British Military), where an appropriate decision could be made around the risk posed. Jordan summarised this by saying:

“We decided to take a vote on it and initially 3 people wanted to go forward and along the ridge and 2 decided it was, probably too risky and I was one who said that is wasn’t as bad as it looked, there was a safe way off…So, I think we made the right call.”

This strategy was employed throughout the challenge while on the mountain to mitigate the risk. Indeed, [27] highlighted the importance of effective communication to maximise team effectiveness and minimise risk within a polar environment. It was also attempted early on in the challenge within the basecamp setting, but as the cluster effect developed and cascaded, it was not fully adhered to, causing a subsequent stressor of social support and individual differences.

*Humour.* In terms of apprising the environment as a challenge, participants emphasised the importance of humour, which was used throughout the challenge. Blair summarised the use of humour during focus group 2:

“I think we just sort of bounce off each other a bit, don’t we? And, you know, try and have a bit of a laugh, if you see someone down, just try and pick them up a bit, you know, sort of we’re always having a laugh and a joke and, you know, it seems to keep everyone’s morale up… it probably releases…tension is not the right word, but I think it just, a little bit of humour goes a long, long way. I think when you’re faced with the challenges that we’re obviously faced with day in, day out, irrespective of the challenge itself. Obviously in addition to all the personal challenges that people are facing, it just sort of, it’s a smile, a bit of humour can make the day a lot, lot brighter. And obviously it needs to because the days are long and they’re only going to get longer, and the challenges are only going to get more and more arduous as we go on.”

Humour has been well-documented as a coping strategy [84,85,86] and contributes to resilience, enabling individuals to cope with extreme environments [3]. It is emotion-focused and looks to cognitively reframe stressors by reducing the severity of them to buffer their impact [3,84]. This was seen on several occasions; for example, during focus group 3, Charlie highlighted an experience while on the mountain:

“Just having that focus to get up each one and again it was just…and yeah we have a right laugh when we’re out it’s a bit ridiculous really some of the things that we’ve done. Look at me. And looking across, I mean, we were on… We were going up one mountain it was the worst one we’ve been and I looked across to Blair and I mean it was literally like that [makes hand gesture about the slope] but it was all just loose stone and shingle and slate and everything else so every time you moved the whole mountain just moved and I’ve look across to them and we were just laughing at each other and I think if you haven’t got that sense of humour you’d kind of knock it on the head.”

Early on in the challenge, Charlie thought humour was important: “A good sense of humour. The team seems to have a good sense of humour. We’re having, although it is brutal, you know, we’re having good fun, good banter”. Kendall also suggested humour was important towards the end of the challenge:

“We actually had a bit of a laugh, just I didn’t really chip in much, but, you know, we all had a laugh…There was a bit of a challenge yesterday and the way I kind of deal with it is to just laugh about it.”

It has been highlighted that humour is a diverse construct with individual differences to how humour is used [86], and that it is not always a positive strategy to use if the humour is misinterpreted [84]. This may have been present during the challenge, whereby the use of humour could have been misinterpreted and perceived differently, leading to increased stress and further complexity of the social support within the challenge and interpersonal differences. This, in turn, increased the strain on the social dynamic within the team, which developed into an additional stressor that needed to be attended to. This misinterpretation may have started before the challenge started where comments made could have been misconstrued. This suggests that members of a team entering an extreme environment should attempt to do so with a metaphorical clean slate in relation to the different personalities and potential social interactions between them. Moreover, highlighting that the preparation phase of any challenge team entering an extreme environment is an important bifurcation point where it is imperative that team members become aware of each other’s personalities and potential strategies they may employ during stressful periods.

*The Complexity of Social Support.* This study demonstrates the complexity of social support within an extreme environment with challenge team members drawing upon a unique mix of perceived and actual received support. The importance of resilience for coping with social stressors and dealing with physiological stressors such as fatigue has been outlined [82]. Therefore, social support is important in extreme environments. Within the 100 Peaks, this support came from different social agents, which was dependent on whether they were out on the mountain, on the bikes, or back in base camp. For example, Charlie bestowed a high level of praise on the support team during the early stages of the challenge:

“All of us really is I would say sorting the base camp out. Within a couple of days they got it running like clockwork for us…So really the support team, at the moment the support team are what’s making this happen for us. I mean we…well, for me, we’ve got the easy job, we’re sort of doing what we love doing…it’s such a hard physical challenge, it is easy for us because we’re not having to come home and cook our tea, wash our clothes, get everything ready, these guys are doing it all for us. So, although the days are long and that, it’s brilliant, it really is.”

During focus group 3, they again highlighted their appreciation for the support team, and that they would rather be on the mountain than deal with the stressors at base camp:

“They’ve been around, they’ve been terrible the midges. If it’s not pissing down with rain and freezing cold then the midges are out but it’s like I’ve said for us we’re up in the mountains or on our bikes so we do get away from it for a long period of time these guys they’re never away from it. The weather’s either shit for ‘em or they’re getting bit too… We come back here and they’ve got nets over their faces and they’re still cooking and getting stuff ready, washing, drying it can’t be easy and like I say it’s not a job… I’d much rather be climbing mountains all day than doing all that.”

These social agents were supportive in some instances and perceived as a stressor in others, indicating that different contexts required different forms of support for different people. Social support is an important to buffer to the stressors in the environment, which influence an individual’s ability to perform and maintain functioning and well-being. However, when social interactions are considered a stressor, they will inhibit an individual’s functioning by contributing to the cluster effect of stressors in the environment (see the interpersonal differences section below). For example, the incorporation of partials into the challenge team for some of the days had both a positive and negative effect. For example, during focus group 2, Blair highlighted the morale boosting effect of being surprised by a respected member of the TABing community, who was named Partial here:

“And on the top of the mountain we had [Partial] sitting there waiting for us with a carrier bag full of snow, stuffed with Trooper [bottled beer] in it. And the bloke had driven 500 miles just to be there and come and TAB with us, which is massive.”

This experience was also alluded to by Kendall during their video diary on the morale boosting effect this had on the whole team, but they also highlighted the potential negative impact:

“[Partial] wasn’t as fit as I was, kind of, left with them, and I encouraged them. I looked after them and I looked after them and the [other challenge team members] went off. So, I that made me pretty pissed off to be honest.”

Furthermore, it was the small gestures that had the most significant impact on the participant’s ability to maintain functioning by boosting morale. Again, during focus group 2, there was a discussion between Charlie and Blair around the actions of one of the support team:

“I mean a prime example was like yesterday, we was in the mountains for a good while. And the conditions were rubbish, you know, rain, wind, couldn’t really see a lot in front of you. And we was up there like yesterday, what, eight/nine hours. You know, and then we come back down and [Support Team Member] is there with a hot chocolate.

He offered us chocolate bars.

Just that, it’s a real sort of morale lifter.

Just, that it’s simple things like that, it really is simple things. When you’ve had a hard day, the thought of actually coming back and seeing that you’ve got something hot and steaming and sweet.”

These small gestures of support appeared to buffer the impact of the cluster effect of the stressors within the environment, and these not only came from physical interactions, but also from messages of support through social media platforms.

*Interpersonal Differences.* The extreme environment intensified the relationships and differences between individuals in the challenge. Hence, a significant everyday stressor were interpersonal differences between individuals, which increased through the challenge contributing to challenge team members ability to function, perform, and maintain wellbeing. These differences contributed to the complexity of social support identified in the previous section, potentially preventing social support being used a strategy to buffer the cluster effect. Interpersonal differences are an inevitable aspect of operating in extreme environments [81], with many contributing factors including a culmination of fatigue during the challenge and existing pre-challenge differences. It has been highlighted that when working in an isolated and confined environments (e.g., Antarctica in their review), there is heightened friction, hostility, and conflict [32]. It was clear that there was a clash of personalities between the members of the challenge and support team. This evolved into a major contributing stressor to the cluster effect [31]. It affected team members’ enjoyment, motivation, and energy and centred around pacing on the mountain, being in base camp, and interactions between the challenge and support team. As [23] stated, an individual’s psychological reaction to operating within an isolated and confined environments can be affected by interpersonal factors. For example, during the last focus group, Blair tried to describe these differences without specifically mentioning them:

“Everybody’s physically tired, mentally tired and I mean we had a lot of days where you’re not so much the stuff that we was doing was possibly physically demanding but it was mentally demanding.”

Personality differences were difficult to deal with, and Blair took time to comprehend personality conflicts and dwelled on these over several days, culminating in their polite articulation of these differences, as seen in the quote above. This cascade over a number of days contributed to the cluster effect, as they had to contend with other stressors that were presented each day, further contributing to the complexity of the situation. These personality differences were present in all the participants’ video diaries, with clashes between different members of the support team, challenge team, and visitors. For example, Charlie found that they held back on occasion from voicing differences, supporting the need for tolerance and flexibility [27].

As some of the relationships between individuals were new and/or developing, it took time for everyone to work each other out. This was highlighted by Kendall during the second focus group:

“You do face challenges like every day actually. When you come onto the hills, you’ve got mud, slime and yeah, like with communication as well. At first you need to, you know, get used to the people. See how they, you know, work and things like that. And the longer the challenge goes on, you know, the better it gets, you get to know each other better. But to pick out a specific challenge, it’s quite hard, because every day, you know, every mile is a challenge, you know, sometimes you’ve got sore legs on the bike. Well, you just have to push through and, you know, work together and help each other.”

And during the third focus group Jordan said:

“You’ve got to accept at the end of the day individuals have different personality traits and it’s trying to get used to how people operate. You’ve gotta then learn how to try and instil the best behaviour part of everybody to ensure that you get where you need to be. And I think with a challenge like this it’s probably very difficult because although it’s a seemingly long period of time it’s not really in the grand scheme of things. 25 days isn’t long to spend with people that you’ve probably never spent 25 days with before to completely understand them as individuals and obviously that takes a long time to work out the kinks but it’s the getting there slowly but surely.”

An aspect of operating in extreme environments is that personal space and privacy is limited [81]. Base camps offered limited personal private space, and thus, tensions were heightened due to the conditions, but also because there was limited free time to employ coping strategies such as past times [32], as time in base camp was needed for vital admin duties (e.g., eating, sleeping, cleaning, and preparing kit). This is shown in a quote from Jordan during the final focus group:

“Probably not spent that amount of time in close proximity with these people before and there’s always gonna be the odd tension that’s gonna spring up from time to time it’s just a case of if that arises putting the team first and thinking “I’ve gotta work with all these people” and getting on with it for the sake of the main goal.”

These interpersonal differences had to be put to aside for the sake of the challenge. This proved difficult due to the cluster effect. This also increased perceptions of isolation [31,87] for some participants. This, alongside unfavourable conditions in base camp (e.g., poor weather), contributed to reduced social interaction between the team and support team, as there was a preconceived notion of what the challenge was going to be like at the end of each day (e.g., sitting around a campfire). If this notion had manifested itself, informal reflections/conversations could have occurred, which may have allowed any differences in opinions to be aired and ironed out.

The results of the present study highlighted the individualised dynamic and temporal nature of psychological resilience within an extreme environment. This was influenced by the unique combinations of stressors within the challenge, which affected the team’s ability to function. These stressors produced a cluster effect that team members had to contend with. This cluster effect then cascaded throughout the challenge, forcing a range of strategies that were used to ensure the challenge was completed.

## 4. General Discussion

The findings of this study extend our understanding of the temporal and dynamic nature of resilience, emphasising the complexity and individualised notion of the phenomena. To our knowledge, this is the first study to explore resilience over time within an extreme environment and the strategies to maintain functioning of individuals operating in them providing an original contribution to the understanding of resilience as an interactive process between the individual and environment. It also advances our understanding of the complexity of social support and how it is used as a strategy to buffer the cluster effect of the unique combination of stressors within the environment. Finally, it has advanced our understanding of how a challenge mindset can be used in an extreme environment by those operating in them. This study has offered an original contribution by extending our understanding of resilience, which was achieved by using novel methods of data collection (e.g., video diaries) within a mixed method approach. These methods allowed resilience to be individually tracked over time using quantitative measures, while acquiring depth and perspective through qualitative exploration while individuals are within an extreme environment.

The current research supports the grounded theory developed by [15]. Namely, that psychological resilience should be considered in relation to the specific environment and is dynamic in nature [21]. This is so that the distinct stressors in terms of quantity, duration, and intensity can be identified and to understand how they cluster together [3]. The exposure to these stressors needs to be buffered and inhibited by appropriate strategies and the use of personal qualities to maintain functioning [14]. Two of the psychological factors proposed by the Grounded Theory of Resilience [15] were specifically identified in the results. These included perceived social support and focus (within a challenge mindset [14]) on small manageable objectives to keep moving towards the long-term objective of the challenge. Social support in the challenge was complex, and when utilised by team members, it had a buffering effect against the cluster effect of stressors. When social support was perceived to be negative, it became a stressor and contributed to the cluster effect. The ability to focus on avoiding distractions on the mountain mitigated risks by focusing on the process rather than the outcome [15], giving the challenge team an element of control.

The results support The Everyday Stress Resilience Hypothesis [20] and the individualised and dynamic context of resilience [21]. An appropriate analogy to use here for the Everyday Stress Resilience Hypothesis is the running of a marathon [20]. A runner would not just go out a run a marathon, but would slowly increase the distance until they could successfully achieve the marathon, while taking into consideration the aspects that could affect their ability to run the 26.2-mile distance (e.g., nutrition, injury management, and logistics to find time to train while balancing other commitments). This is also the case with the challenge team; they had to slowly build up their training to allow them to successfully complete the challenge. This slow inoculation must be typical and not chronic in nature, as this would provide setbacks to the person to allow the building blocks to be established, similar to the marathon runner example.

The relationship between how the individual interacts with the environment they are in with continual exposure to those stressors causes the individual to adapt and become inoculated to buffer their impact [88]. However, the interaction is not just dyadic; there is also influence between others in the environment and across different environments in which the individual resides (as seen in the complexity of social support and interpersonal sections, see above). This points to a systems theoretical perspective to be adopted. One such perspective is the Ecology of Human Development model [19] that could be used to explore the interaction of the individual in a complex dynamic environment and how their resilience changes over time and throughout different contexts. Therefore, resilience is the process not only of the dyadic interaction between the individual and their immediate environment, but of the interaction with other environmental stressors within connected environments. These combine to produce a cluster effect that needs to be effectively buffered using appropriate protective factors at the individual’s disposal (e.g., psychological qualities and/or social support). This is because resilience develops in individuals due to intermittent and frequent experiences of different stressors at different times to varying degrees in a complex and ever-changing environment. This is how the individual perceives the stressors in the environment and how they regulate this stress, which demonstrates resilience; it is the interaction between the individual and the environment [16]. Therefore, people positively adapt to the stressors, with resilience emerging, because this adaptation provides an increased capacity to cope when future stress is experienced [20]. This posits that a lifespan longitudinal research methodology should be applied to future research projects.

### 4.1. Strengths and Limitations

The current research design provides an innovative and flexible method of data collection that allows the exploration of psychological resilience longitudinally and ‘live’ within extreme environments, with the use of video diaries offering a viable method of exploring complex phenomena such as resilience. As suggested in other contexts, the use of video diaries should be used in conjunction with other methods to provide a deeper understanding of the complexity of functioning in extreme environments. This supports another strength of the current study; the use of mixed methods to explore resilience over time. The quantitative measures allowed individual differences to be tracked over time, while the qualitative methods allowed individual perspectives of resilience and how functioning was maintained during the challenge to be explored in depth. For example, the focus groups allowed greater depth to be gathered around the collective experiences of the challenge team, highlighting the complexity. They also acted as an opportunity for the researcher to become more immersed into the challenge, which is a key tenant of IPA [44].

However, the video diaries themselves could have served as a resilience strategy to maintaining functioning by merely allowing challenge team members to reflect and verbalise their experiences [16]. Nevertheless, despite the study’s success in exploring resilience over time, there were still some concerns about whether the video diaries captured resilience live, as they were completed as close to the event as possible. As a result, there could still be elements of recall bias, as the participants were reflecting on the event after it had occurred. Due to the time constraints of the challenge, the video diaries were often completed as close to the event as possible, and not always at the end of each day. This could be interpreted as a demonstration of the cluster effect, as challenge team members were required to attend to relevant stimuli needed to maintain functioning. This was due to the vital tasks that needed to be completed, and it was often the video diaries that were the first to be dropped from daily task lists, as they were considered non-vital.

Due to the social dynamics within the challenge team members, they may not have given their honest opinion and might have held back some of their comments during the focus groups, preventing depth to be achieved. Moreover, they could have been giving responses they thought the researcher wanted to hear. Finally, the focus groups were largely completed in remote and unfamiliar locations, so the challenge team may not have been physically comfortable as well as having other tasks to complete around base camp. This may have caused them to be distracted and uncomfortable, which caused them to give shorter/incomplete answers, so they could attend to other essential administration duties that they were required to do. With regards to the CD-RISC10, one of the participants, Charlie, scored full points across every time point. This could have been due to them having just ticked the questionnaire without consideration, or it could be that Charlie simply demonstrated high levels of resilience. The CD-RISC10 has previously been used in sport [64,65], but not specifically in its entirety in an extreme environment. Consequently, this measure could have a ceiling effect that does not have measurement sensitivity to effectively distinguish between an individual’s level of resilience in extreme environments. Despite an attempt to not interfere and limit the impact of the research within the challenge, the fact that the lead researcher attended different base camps to complete the focus groups as well as staying in base camp may have affected the team members, and thus, influenced the content they provided in both the video diaries and focus groups.

### 4.2. Future Research

In terms of future research, the current research points towards exploring resilience in other extreme environments due to the unique combination of stressors within each environment. Additionally, the research has highlighted the need to explore resilience over time using a lifespan perspective. Future studies should endeavour to utilise a lifespan perspective to develop an understanding of participants background in relation to past experiences (significant life events and experiences within extreme environments), as these can shape and dictate how resilience may present itself when confronted with the complex array of stressors in a specific extreme environment and subsequent transfer to other life contexts [16].

When exploring resilience methods that incorporate both live and retrospective data, different collection methods should be utilised. This could be achieved by using mixed methods at all stages of data collection. Quantitative measures could track changes in resilience and qualitive methods could explore these individual changes over time. To do this effectively, future research designs need to allow a period of reflection to allow participants to attempt and make sense of what has happened. This is because time (and resilience) is individually perceived, so any changes may occur post challenge and, thus, not captured live within the extreme environment. Any adaptation may occur after the challenge has completed. To do this, there needs to be a push to be creative and innovative in devising ways to do this not only in extreme environments but also other contexts.

### 4.3. Practical Implications

The results from this study have application to those working in and preparing for entering an extreme environment. Awareness should be given to the cluster effect and the strategies used to buffer its potential impact. People entering extreme environments should become as aware as possible of the possible stressors, how they might cluster, and the severity of them. This points to preparing correctly in every possible aspect of the environment (for example, this may be physical, psychological, and social). Despite this, nothing can substitute gaining experience of the actual environment in which an individual will be operating. This should be initially done in small doses to allow those operating in them to become accustomed to the stressors and how they might cluster. There also has to be an emphasis on highlighting the potential for the unique combinations of the stressors in the cluster effect in terms of frequency, duration, and order. The results demonstrate the importance of every member of a team operating in an extreme environment to commit to the long-term objective to enhance the challenge mindset of individuals. Whilst an awareness of the long-term objective is imperative, individuals should break down this objective down into smaller specific and manageable objectives to allow progress to be made toward this long-term objective, so that individuals can put “one foot in front of the other”. The results of this study can also be applied to other contexts. Despite being completed in an extreme environment, the results can be applied to contexts that are less extreme, as every environment will have stressors that have the potential to cluster together. If individuals are not aware of these stressors and how they might cluster together, then functioning and performance could be impaired. The notion of preparation and gaining experience of stressors within the environments that individuals operate in is universal, whether that be in elite sport, health professions, or business. The notion of committing to a long-term goal and then breaking these in short-term targets such as those used by the challenge team also has universal application.

## 5. Conclusions

The current study is significant since it enhances our understanding of psychological resilience within extreme environments. Specifically, it adds to our understanding of resilience as a process, as it emphasises the complex, dynamic, and individualised nature of resilience and how individuals maintain functioning while operating within extreme environments. Specifically, a challenge mindset and social support were employed, but these were used to differing levels and at different times by team members. It has highlighted the importance of being aware of the potential stressors and how they might cluster together. The study is original since it has explored resilience over time within an extreme environment. Methodologically, this study offers a creative and original way to explore psychological resilience “live” and over time to unpick the complex interactions between individuals and the environment from a resilience perspective.

## Figures and Tables

**Table 1:** A summary of qualitative results from the video diaries and focus groups (part 1).

Identification of Stressors	
Significant Stressors	Personal Administration Errors
	Unpredictable Disruptive Incidents
Everyday Stressors	
Cluster Effect	
The Start of the Cluster Effect	
Different Stages and Bifurcation Points	

**Table 2:** A summary of the qualitative results from the video diaries and focus groups (part 2).

Exploration of Resilience	
Challenge Mindset	Acceptance
	Putting One Foot in From of the Other
	Humour
The Complexity of Social Support	
Interpersonal Differences	

## Data Availability

Due to the size of the data files and to maintain confidentiality of the participants, the research data are not available.
